# Pollinators and Other Flying Insects inside and outside the Fukushima Evacuation Zone

**DOI:** 10.1371/journal.pone.0140957

**Published:** 2015-11-11

**Authors:** Akira Yoshioka, Yoshio Mishima, Keita Fukasawa

**Affiliations:** Center for Environmental Biology and Ecosystem Studies, National Institute for Environmental Studies, 16–2 Onogawa, Tsukuba, Ibaraki, 305–8506, Japan; Monash University, AUSTRALIA

## Abstract

Following the accident at the Fukushima Daiichi nuclear power plants in 2011, a large evacuation zone was imposed in an area where residents had historically managed forests and farmlands. Thus, the human activities that had maintained biodiversity and ecosystem services in the zone were discontinued. Such change can affect insects, a biodiversity component that is relatively tolerant to radiation exposure. In this study, we investigated flying insects, including pollinators, important ecosystem providers inside and outside the zone, using Malaise traps. The results showed that the number of individuals of *Xylocopa appendiculata*, the largest Apidae species in the region, was significantly lower inside the evacuation zone than outside it, whereas those of other insects were not lower significantly. Although we suggest that flying insects and their ecosystem services (i.e., benefits from them such as pollination) 3 years after the disaster were not critically impacted, it is important to monitor the long-term effects of the evacuation in the future.

## Introduction

Although nuclear power plants are often believed to provide an attractive option for mitigating climate change [1, 2], accidents at these plants can result in the long-term evacuation of residents over a wide area. A serious nuclear accident at the Fukushima Daiichi power plants in 2011, which occurred in an area with a higher human population density than that surrounding Chernobyl [3, 4], was followed by the evacuation of about 81,000 people [5]. According to estimates made by the national government of Japan, it will take more than 20 years of natural decay for the radiation to reach levels that will permit a complete return of the residents [6]. Decontamination and reconstruction of the evacuation zone are necessary as soon as possible in such cases, and the direction of such reconstruction must be carefully considered. Historically, most of the evacuation zone in Fukushima was covered by forests and farmlands that were traditionally managed [7], and it was assumed that the residents benefitted from biodiversity and ecosystem services in a sustainable manner. In such situations, healthy biodiversity and ecosystem services are highly important components of successful reconstruction and should be appropriately monitored and evaluated.

It has been hypothesised that not only radiation, which has often been the focus of previous studies in Fukushima and Chernobyl [8–11], but also the cessation of the disturbances caused by anthropogenic activities such as farming and gardening seriously affect the biodiversity and ecosystem services in evacuation zones. A meta-analysis of farmland abandonment suggests that this phenomenon has frequently had a negative effect on biodiversity in Africa, Asia, and Europe [12], where the history of anthropogenic land use is relatively long. In Japan, traditional farming that is subject to intermediate disturbances has formed highly heterogeneous landscapes; these have been reported to support high levels of biodiversity and sustainable ecosystem services [13, 14].

Among the components of biodiversity, some taxonomic groups, such as insects, are relatively tolerant of radiation exposure [15–17], and serious effects of exposure to radiation within the Fukushima evacuation zone are unlikely to be found at the population level [11]. A previous study that surveyed the northwestern area of the Fukushima nuclear power plants immediately (6 months) after the accident suggested that the reduction in the abundance of insects exposed to radiation was less obvious than that it was at Chernobyl [10]. However, studies in farmland areas have suggested that insects are sensitive to changes in land use [14, 18] and that these effects can become more obvious with time. Considering the fact that insects play an important role in ecosystem functions and serve as pollinators, pests, and prey for upper trophic levels, such as birds and mammals, it is critical to effectively monitor and evaluate their populations in the Fukushima evacuation area to guide effective reconstruction.

**Fig 1:**
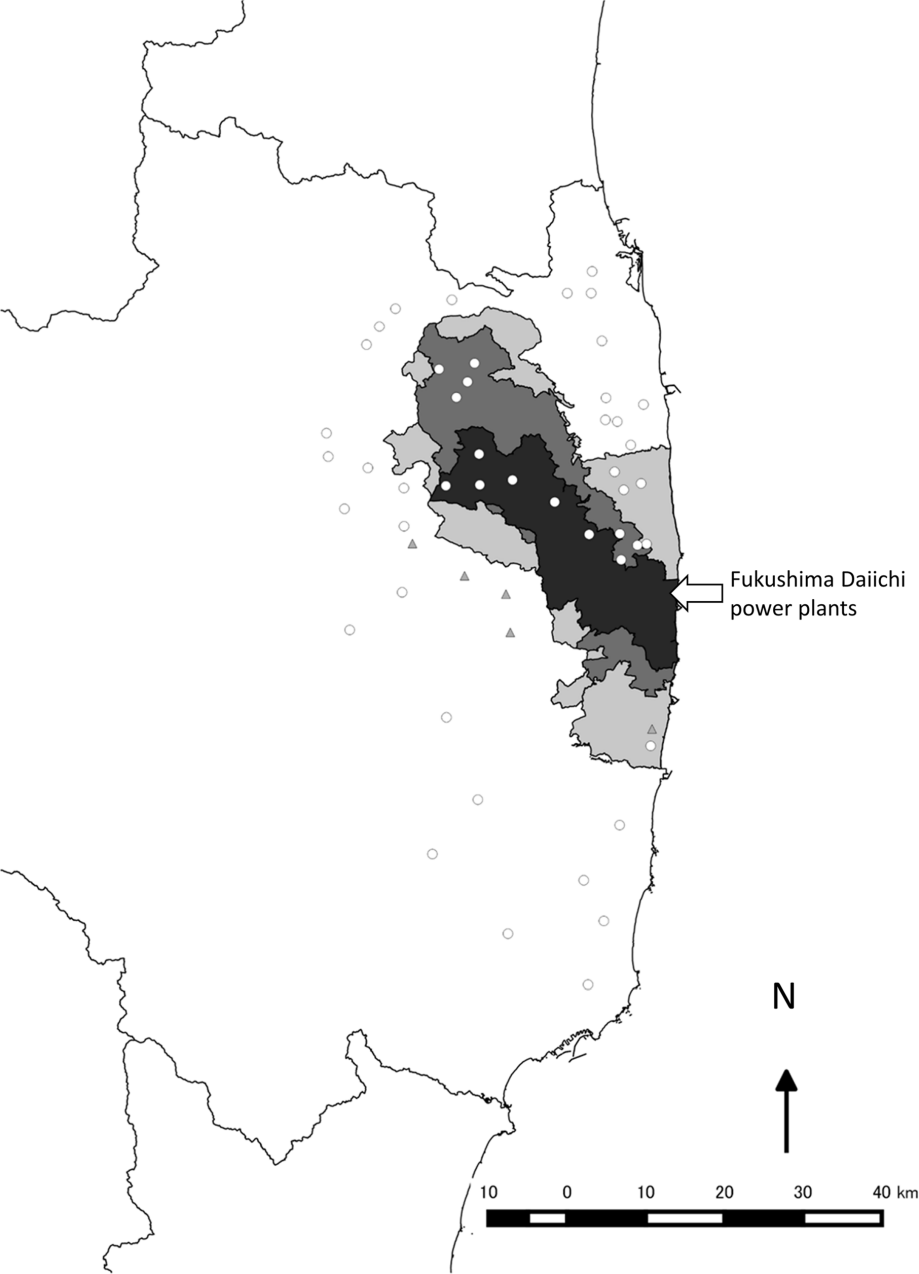
Map of sampling sites. Open circles and filled triangles correspond to sites analysed and not analysed due to disturbances to the traps, respectively. Dark grey, grey, and light grey zones correspond to the subzone preparing for the lifting of the evacuation order, restricted residential areas, and the subzone to which it was difficult to return, respectively. The Coast line and prefectural boundaries were obtained fromOpenStreetMap.

In this study, we investigated differences in the abundances of insects inside and outside the Fukushima evacuation zone (Fig 1) 3 years after the nuclear accident. The data from this study are expected to provide a valuable contribution to efforts to monitor and evaluate biodiversity and ecosystem services in the evacuation zones resulting from nuclear power plant disasters. Specifically, we focused on flying insects, including important pollinators such as Apidae, and medically important pests, such as flies and hoverflies. We covered broad areas and examined taxonomic groups that had not been previously investigated in this region but that are nonetheless important for the provision of ecosystem services.

## Results

**Fig 2:**
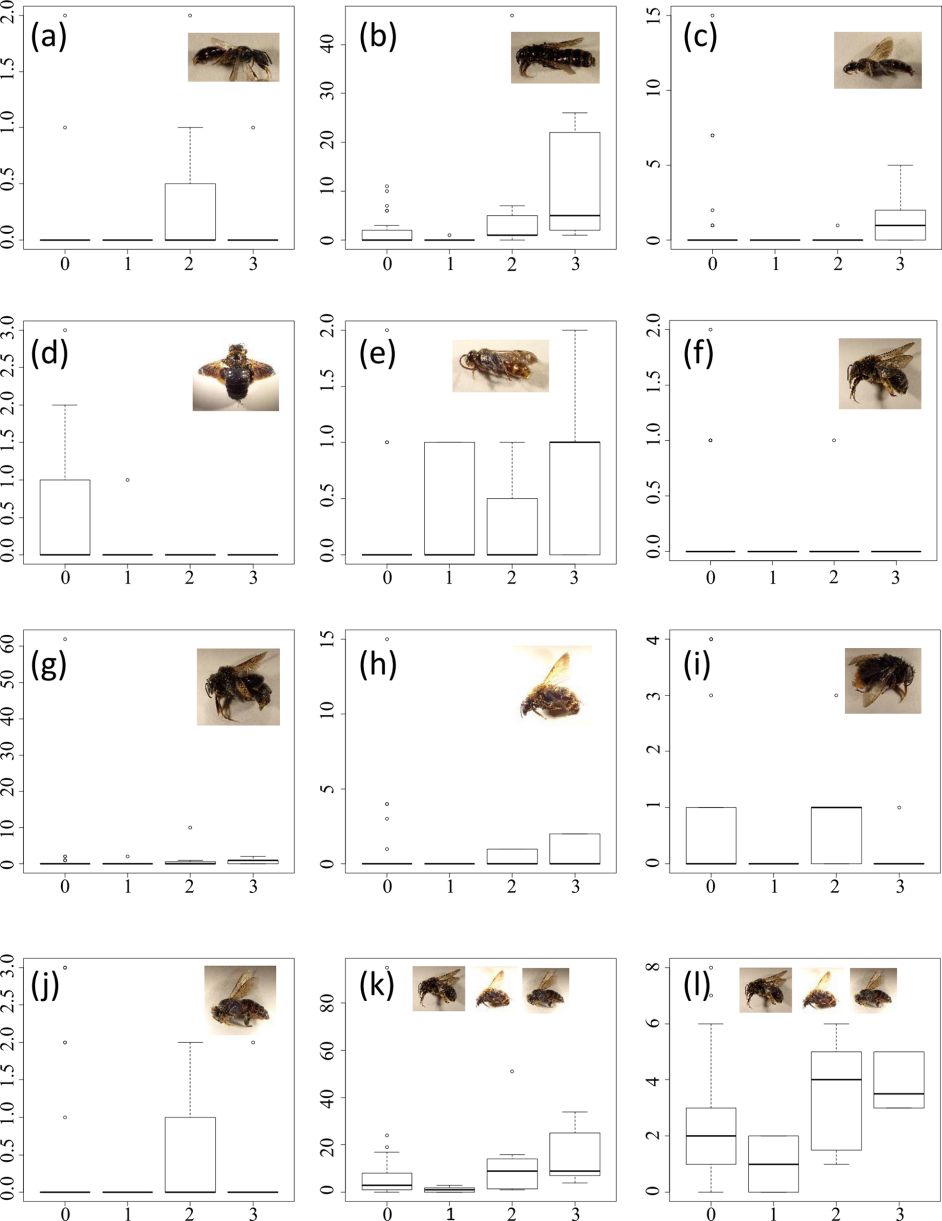
Abundances of (a) *Ceratina iwatai*, (b) *Ceratina flavipes*, (c) *Ceratina japonica*, (d) *Xylocopa appendiculata*, (e) *Nomada* spp., (f) *Eucera spurcatipes*, (g) *Eucera nipponensis*, (h) *Bombus diversus*, (i) *Bombus ardens*, (j) *Apis cerana*, (k) total Apidae, and (l), species richness of Apidae (min) by evacuation level. The vertical axes correspond to the number of individuals. The box plots represent the 75th, 50th (thick horizontal line), and 25th percentiles; the top error bar ranges from the 75th to the most extreme data point which is no more than 1.5 times the interquartile range from the box, and the bottom error bar from the 25th to the lowest data point which is no less than 1.5 times the interquartile range from the box. Small circles represent outlier values from the error bars. Evacuation levels 0, 1, 2, and 3 in the horizontal axes correspond to outside the evacuation zone, the subzone preparing for the lifting of the evacuation order, restricted residential areas, and the subzone to which it was difficult to return, respectively. Note that the species richness of Apidae (max) was omitted.

**Fig 3:**
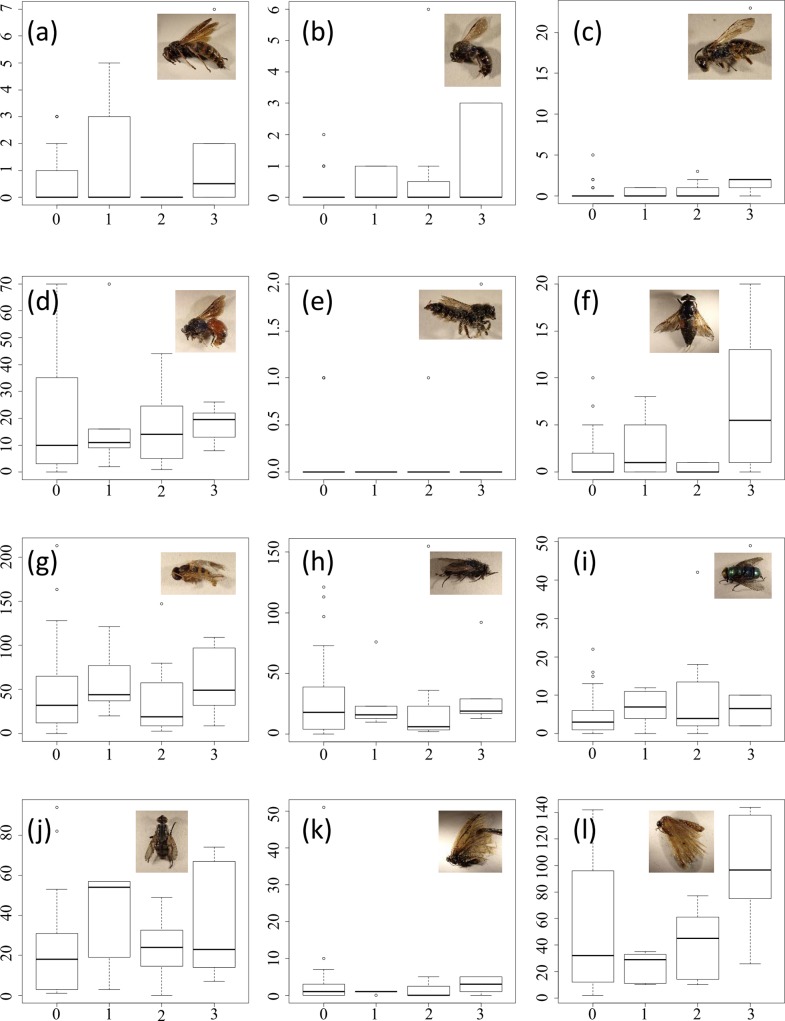
Abundances of (a) Vespidae, (b) Colletidae, (c) Andrenidae, (d)Halictidae, (e) Megachilidae, (f) Tabanidae, (g) Syrphidae, (h) Muscidae, (i) Calliphoridae, (j) Sarcophagidae, (k) butterflies, and (l) moths by evacuation level. The vertical axes correspond to the number of individuals. The box plots represent the 75th, 50th (thick horizontal line), and 25th percentiles; the top error bar ranges from the 75th to the most extreme data point which is no more than 1.5 times the interquartile range from the box, and the bottom error bar from the 25th to the lowest data point which is no less than 1.5 times the interquartile range from the box. Small circles represent outlier values from the error bars. Evacuation levels 0, 1, 2, and 3 in the horizontal axes correspond to outside the evacuation zone, the subzone preparing for the lifting of the evacuation order, restricted residential areas, and the subzone to which it was difficult to return, respectively.

**Fig 4:**
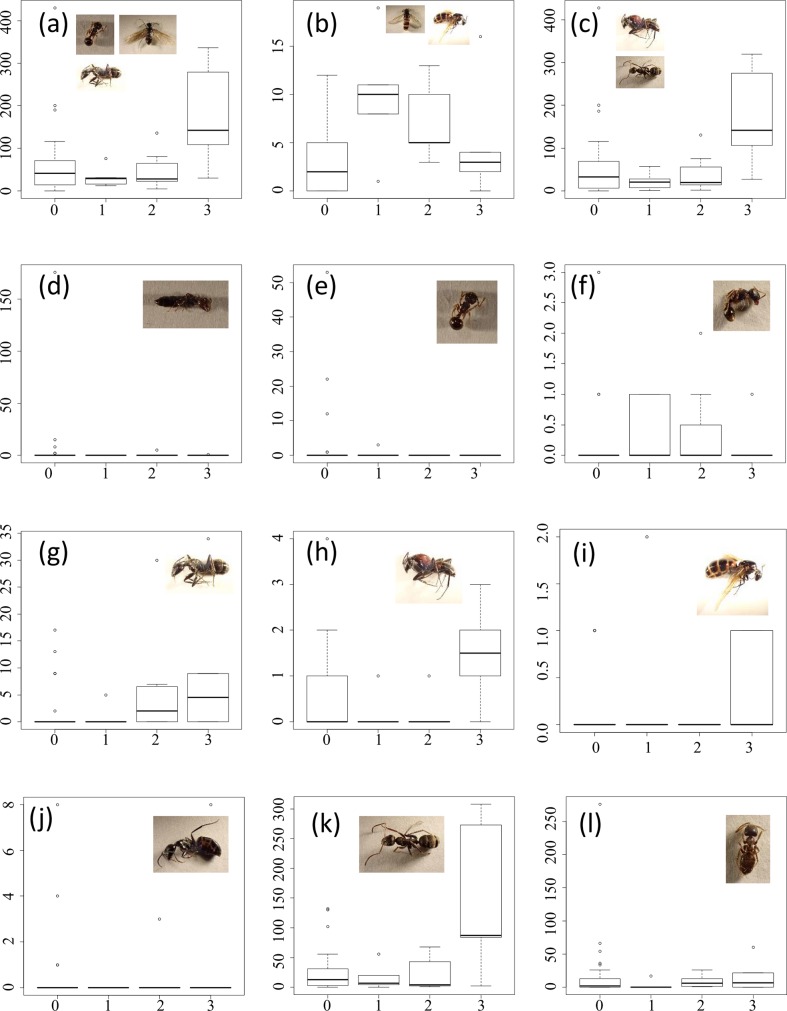
Abundances of (a) Formicidae, (b) total winged ants, (c) total workers, (d) workers of *Crematogaster matsummurai*, (e) individuals of *Pristomyrmes pungens*, (f) workers of *Tetramorium tsushimae*, (g) workers of *Camponotus japonicus*, (h) workers of *Camponotus obscuripes*, (i) queens of *Camponotus obscuripes*, (j) workers of *Camponotus quadrinotatus*, (k) workers of *Formica japonica*, and (l) workers of *Lasius japonicas* by evacuation level. The vertical axes correspond to the number of individuals. The box plots represent the 75th, 50th (thick horizontal line), and 25th percentiles; the top error bar ranges from the 75th to the most extreme data point which is no more than 1.5 times the interquartile range from the box, and the bottom error bar from the 25th to the lowest data point which is no less than 1.5 times the interquartile range from the box. Small circles represent outlier values from the error bars. Evacuation levels 0, 1, 2, and 3 in the horizontal axes correspond to outside the evacuation zone, the subzone preparing for the lifting of the evacuation order, restricted residential areas, and the subzone to which it was difficult to return, respectively.

**Fig 5:**
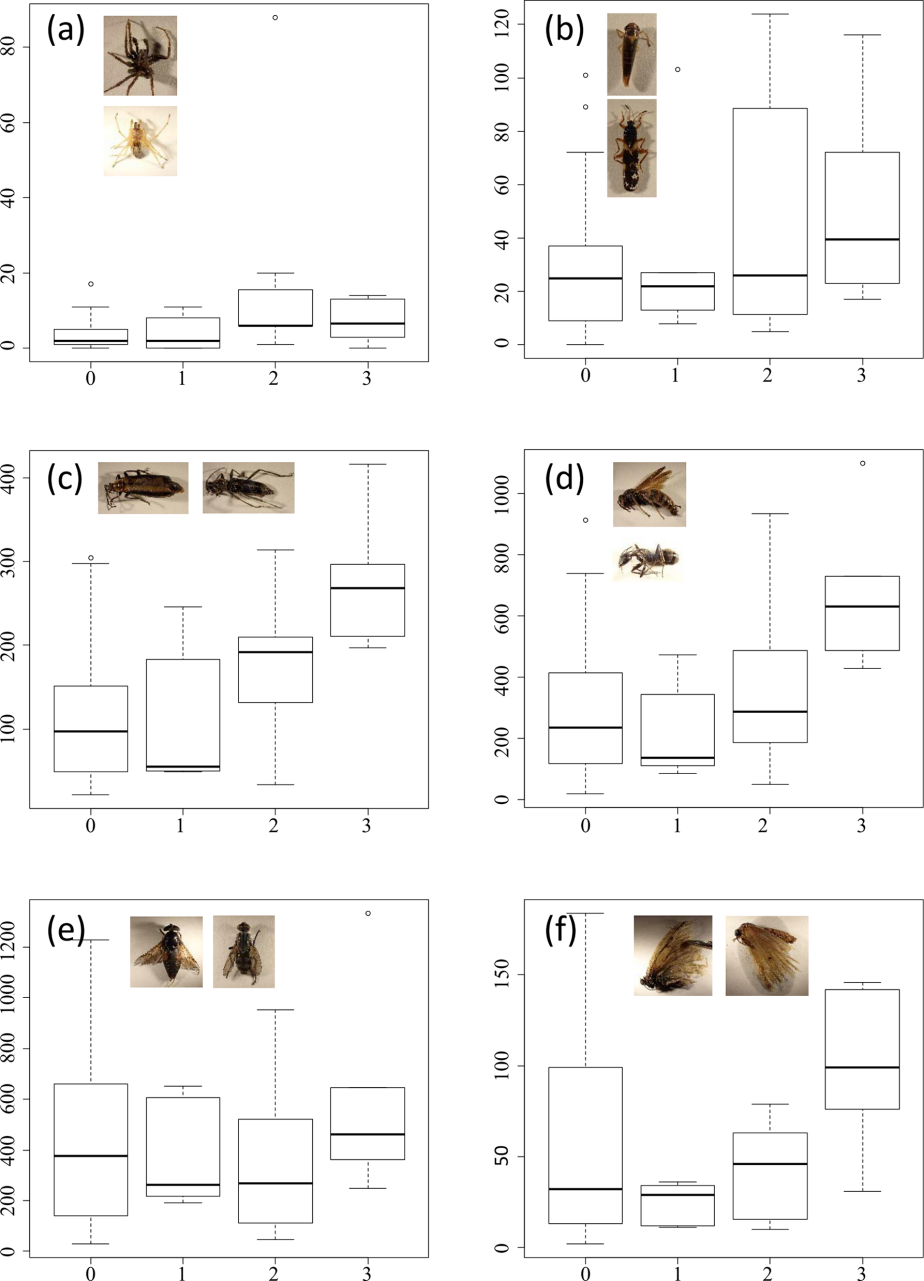
Abundances of (a) Araneae, (b) Hemipteran, (c) Coleoptera, (d) Hymenoptera, (e) Diptera, and (f) Lepidoptera by evacuation level. The vertical axes correspond to the number of individuals. The box plots represent the 75th, 50th (thick horizontal line), and 25th percentiles; the top error bar ranges from the 75th to the most extreme data point which is no more than 1.5 times the interquartile range from the box, and the bottom error bar from the 25th to the lowest data point which is no less than 1.5 times the interquartile range from the box. Small circles represent outlier values from the error bars. Evacuation levels 0, 1, 2, and 3 in the horizontal axes correspond to outside the evacuation zone, the subzone preparing for the lifting of the evacuation order, restricted residential areas, and the subzone to which it was difficult to return, respectively.

Through sampling insects inside and outside the Fukushima evacuation zone in early summer, we found that the number of individuals of *Xylocopa appendiculata* was remarkably lower inside the evacuation zone, whereas those of other insects were not. In more detail, a total of 48,081 insects and spiders in 47 sampling sites were sampled using Malaise traps set from mid-May to mid-July 2014. Most were Hymenoptera and Diptera (16,583 and 20,082, respectively). The mean±SD per site of recorded individuals in each taxonomic and caste group are shown in S1 Table. In total, 46 taxonomic and caste groups (6 orders, 10 families, butterflies, moths, 12 species and 5 genera of bees and wasps, total wing ants, total workers of ants, workers of 8 ant species including a polygynous species, and queens of 1 ant species) and the species richness of Apidae were analysed (Tables 1–3; Figs 2–5; S2 Table, S1 Data).

**Table 1:** Medians and credible intervals of the partial regression coefficient for exclusion "*b*_*0*_" on Bayesian statistical models for number of individuals and species richness of Apidae species.

Taxnomic group	2.5%	25%	Median	75%	97.5%
**Apidae**	-0.311	0.470	0.883	1.287	2.124
*Ceratina iwatai*	-5.563	1.792	4.689	7.669	14.252
*Ceratina flavipes*	0.682	1.933	2.602	3.420	5.172
*Ceratina japonica*	-5.622	-2.135	-0.681	0.745	4.426
*Xylocopa appendiculata*	-20.241	-10.790	-6.956	-4.420	-1.663
*Nomada spp*.	0.564	1.823	2.572	3.432	5.711
*Eucera spurcatipes*	-13.692	-5.451	-2.455	-0.204	4.407
*Eucera nipponensis*	-2.714	0.304	1.628	3.079	6.702
*Bombus diversus*	-4.245	-0.625	0.868	2.540	6.812
*Bombus ardens*	-5.694	-1.654	-0.289	0.938	3.892
*Apis cerana*	-3.427	0.022	1.274	2.651	6.716
^1^ **Species richness of Apidae (max)**	-0.085	0.374	0.591	0.820	1.289
^2^ **Species richness of Apidae (min)**	-0.131	0.339	0.564	0.792	1.264

^1^Species richness of Apidae assuming that unidentified Ceratina sp. was allospecific to individuals indentified to species levels in the site.

^2^Species richness of Apidae assuming that unidentified Ceratina sp. was conspecific to individuals indentified to species levels in the site.

**Table 2:** Medians and credible intervals of the partial regression coefficient for exclusion "*b*_*0*_" on Bayesian statistical models for number of individuals of the other pollinators and/or medically important pests, and ants.

Taxnomic group	2.50%	25%	Median	75%	97.50%
**Vespidae**	-4.608	-2.023	-1.076	-0.209	1.628
*Polistes snelleni*	-5.020	-2.315	-1.266	-0.336	1.471
**Colletidae (*Hylaeus* spp.)**	-1.717	0.743	1.714	2.718	5.655
**Andrenidae (*Andrena* spp.)**	-0.075	1.380	2.086	2.946	4.936
**Halictidae**	-0.892	-0.218	0.099	0.445	1.108
*Halictus aerarius*	-2.168	-0.230	0.561	1.381	3.573
*Lasioglossum occidens*.	-2.667	-0.531	0.479	1.529	3.665
*Lasioglossum* spp.	-0.941	-0.249	0.081	0.396	1.093
*Sphecodes* spp.	-4.892	-0.533	1.143	2.797	8.047
**Megachilidae**	-9.408	-2.501	-0.510	0.865	4.807
**Formicidae**	0.094	0.842	1.286	1.719	2.478
**Total winged ants (queens and males)**	0.008	0.682	1.034	1.357	2.093
**Total workers**	-0.018	0.940	1.464	2.028	3.030
Workers of *Crematogaster matsumurai*	-13.900	-6.210	-2.516	1.011	8.374
Individuals of *Pristomyrmex pungens*	-23.370	-13.650	-9.200	-5.496	1.734
Workers of *Tetramorium tsushimae*	-4.420	-0.420	0.986	2.586	6.922
Workers of *Camponotus japonicus*	0.903	3.553	4.989	6.658	10.532
*Queens of Camponotus obscuripes*	-7.011	-0.197	2.055	4.547	11.481
Workers of *Camponotus obscuripes*	-1.961	-0.398	0.318	1.104	3.066
Workers of *Camponotus quadrinotatus*	-12.320	-3.714	-0.066	3.505	11.430
Workers of *Formica japonica*	0.548	1.806	2.404	2.981	4.103
Workers of *Lasius japonicus*	-2.079	-0.232	0.614	1.575	3.573
**Tabanidae**	-0.866	0.458	1.144	1.874	3.304
**Syrphidae**	-0.844	-0.063	0.299	0.682	1.454
**Muscidae**	-0.810	-0.017	0.372	0.776	1.688
**Calliphoridae**	-0.563	0.310	0.698	1.130	2.015
**Sarcophagidae**	-0.318	0.452	0.857	1.287	2.143
**Butterflies**	-1.507	-0.494	0.011	0.558	1.770
**Moths**	-0.371	0.186	0.485	0.815	1.462

**Table 3:** Medians and credible intervals of the partial regression coefficient for exclusion "*b*_*0*_" on Bayesian statistical models for number of individuals of each orders.

Taxnomic name	2.50%	25%	Median	75%	97.50%
**Araneae**	-0.058	0.726	1.106	1.476	2.219
**Hemiptera**	-0.478	0.114	0.392	0.678	1.257
**Coleoptera**	0.369	0.757	0.960	1.185	1.655
**Hymenoptera**	0.048	0.513	0.798	1.089	1.650
**Diptera**	-0.510	0.033	0.309	0.613	1.289
**Lepidoptera**	-0.371	0.193	0.511	0.807	1.466

Bayesian statistical analysis was used to assess the effect of evacuation on each taxonomic group. In the analysis of *Xylocopa appendiculata*, a species of carpenter bee of the Apidae family, the upper limit of the 95% credible interval (CI) regression coefficient for the evacuation effect, *b*_*0*_, was <0 because few individuals were captured in the evacuation zone (Table 1; Fig 2D). Although the European honey bee *Apis mellifica* was captured in the evacuation zone, the number of sites in which they were present was too small (three points) to construct a statistical model. In contrast, *Ceratina flavipes* and *Nomada* spp. showed remarkable positive effects from the evacuation (the 95% lower limits of CI of their coefficients for *b*_*0*_ exceeded 0) (Table 1; Fig 2B and 2E). The species richness of Apidae showed no remarkable effects from the evacuation when both cases of the *Ceratina* sp. were counted as one species and when they were counted as multiple species (Table 1).

No remarkable negative effects from the evacuation were shown for the other taxonomic groups (Tables 2 and 3; Figs 3–5), whereas Coleoptera, Hymenoptera, winged ants, and workers of *Camponotus japonicus* and *Formica japonica* showed remarkable positive effects (Tables 2 and 3; Fig 4B and 4G; Fig 5C and 5D).

## Discussion

We found several notable differences inside and outside the evacuation zone with respect to various flying insects, including pollinators, 3 years after the power plant accident, even when we incorporated spatial autocorrelation and the effects of surrounding landscapes before the disaster. Statistical modelling at the species level in Apidae suggested that the density of *Xylocopa appendiculata*, the largest Apidae species in the region, was significantly low in the evacuation zone, whereas the densities of small species, such as *Ceratina flavipes* and *Nomada* spp, were higher. Bumble bees were not affected by the evacuation, which is consistent with the rapid census conducted by Møller et al. [10] 6 months after the disaster. In addition, modelling at a higher taxonomic level in other insects suggested that the majority of insects did not significantly differ by evacuation (or were present at a higher density). However, it should be noted that we did not differentiate species composition.

Considering previous studies on insects and radiation levels (of less than several dozen micro greys per hour) in the evacuation zone [11, 16], it is unlikely that the radiation directly damaged the population of bees. In particular, the life history of the carpenter bee suggests that, unlike bumble bees, which often nest underground, it is not susceptible to exposure to high levels of radiation [19, 20]. Alternatively, the potential cause of the lower density of the carpenter bee in the evacuation zone might be the reduction in the flowers of garden plants following the evacuation. *Xylocopa* are generally found in tropical zones [21], and the bees also favour large and conspicuous flowers such as wisterias, orchids and passion fruits [22–24] as well as gardenias.

The higher density of small bees and other insect groups may be due to “release of resources” by the cessation of human activities such as farming. However, caution is required in interpreting the results because our sampling method, which relied on Malaise traps, was not optimal for worker and wingless ant species. It is worth noting that *Ceratina flavipes*, which prefers lowland areas [25], were found at higher density in the evacuation zone, which is in contrast to the distributions of other congeneric species. The cessation of farming, which had generally been dominant in the lowland areas of the evacuation zone, may have increased the wild plants that provide food and nesting materials for this species. The positive effects on *Nomada* spp. should also be interpreted with caution because they occurred at a low density and as multiple species. A potential ecological cause of the observed pattern of *Nomada* spp. might be that, as one of the smallest species groups in Apidae in Japan, *Nomada* spp. could make use of a broader range of flower species, including Compositae weeds, with relatively small flowers [25], which are often found in abandoned lands [26, 27].

Fortunately, there appeared to be no marked loss of ecosystem functions and services by flying insects in the evacuation zone. Pollinators (bees, wasps, butterflies, and hoverflies) were shown to be abundant, with the exception of the absence of carpenter bees, whose importance for ecosystem services is relatively low in temperate zones [21, 22]. In addition, re-colonisation of the species after reconstruction might not be difficult because large bees, such as bumble bees, have been observed to show high dispersal ability and rapid range expansion [28, 29]. The abundance of medically important pests, such as flies and horseflies, tended to be higher, but not significantly so, differing from the higher rates observed in the coastal areas of northern Japan immediately after flooding following the tsunami [30, 31].

However, the long-term cessation of anthropogenic activities and the resulting disturbances may negatively affect certain insect groups, or groups not evaluated in this study (e.g., aquatic insects that depend on rice paddies), in the future. Moderate disturbances from farming have been reported to have a widespread positive effect on biodiversity in Japan [13, 14, 32]. Therefore, continuous monitoring and complementary studies will be needed to effectively plan the return of residents to the evacuation zone in a way that incorporates biodiversity and ecosystem services. Future monitoring of not only insects but also changes in land use (and radiation levels) following reconstruction will also be able to demonstrate if our results, which were derived from snapshot data, were merely historical effects existing before the evacuation.

Nevertheless, our sampling method, which was relatively labour-saving but covered broader areas using socially-stabilised sampling locations (i.e., schools and community centres), favoured indicator species that commonly occurred around the disaster area and are sensitive and/or related to human activities (e.g., carpenter bees). Thus, they provide a good basis for a biodiversity monitoring system in the evacuation zone. Considering the fact that the number of nuclear power plants will increase in Asia [2], where the history of land use is relatively long [7, 33] and the population is still growing [34], our research will be of benefit efforts not only to reconstruct the Fukushima site but also to effectively respond to future potential disasters.

## Materials and Methods

The local governments in Fukushima prefecture, Japan (Date city, Iitate village, Iwaki city, Minami souma city, Namie town, Naraha town, Nihonmatsu city, Souma city, and Tamura city) granted permission for the Field study.

### Study area

Sampling was conducted in 2014 at 52 sites covering 9 municipalities, including the evacuation zone around the Fukushima Daiichi power plants (Fig 1). The sites outside the evacuation zone were selected to enclose the zone so that they adequately included the regional insect communities and to prevent spatial variations in the insect communities from masking the effects of the evacuation.

Under permission by the corresponding local governments, all the sampling sites were placed within schoolyards (including those that had been converted to community centres), which minimised differences in the local conditions of the sites.

### Sampling and sorting

A large tent-like Malaise trap (1.65 m in length, 1.8 m in width, and 1.8 m in height) with a 500-ml sampling bottle (EZ-malaise trap BT1002, MegaView Science Co., Ltd, Taichung, Taiwan) was set in each sampling site from mid-May to mid-July, when various flying insect species are expected to emerge and function as prey for mammals and birds. We added 250 ml 50% neutral dish detergent pre-diluted with Millipore water to the sampling bottle of each trap in advance. Some of the sampling bottles were replaced in mid-June, depending on the volume of captured insects. In five sites, the traps had fallen over, and sampled insects had spilled out of the bottle. Therefore, the samples from only the remaining 47 sites were used in the analyses.

The samples captured in the traps were transferred to the laboratory and stored in 99% ethanol. Subsequently, all insects and spiders with a body length larger than around 4 mm (this threshold covers all Apidae species in Japan [25]) were sorted and counted, focusing on pollinators and medically important pests. As for ants, however, species with a body length smaller than 4 mm were also sorted and counted. Adult Hymenoptera individuals, one of the main targets of the traps, could be relatively easily identified, and were classified as bees, wasps, ants, or others. Then, bees, wasps, and ants were sorted into genera or species, if possible. Winged ants (i.e., queens and males) were also counted. Adult Diptera individuals were classified as hoverflies (Syrphidae), pollinators, Tabanidae, Muscidae, Calliphoridae, Sarcophagidae, medically important pests, or others. Adult individuals of Lepidoptera were classified as butterflies or moths. Araneae, Hemiptera, and adult Coleoptera were sorted according to order. Individuals in other taxonomic groups or growth stages were not classified because they were assumed to be too sensitive to local environments instead of to the broad-scale changes of surrounding landscapes. Nomenclature for Hymenoptera followed Terayama [35]. Data of the taxonomic groups which were unambiguously classified and presented more than 5 sites were used in the subsequent analyses. In addition, the species richness of Apidae was also analysed because almost all Apidae species were unambiguously classified. Note that species of *Nomada* spp. were not adequately identified, but the number of individuals was small and only one species was present in each site. In addition, only one individual of *Ceratina* sp. was not identified to the species level.

### Environmental factors

The Fukushima evacuation zone was categorised into three subzones based on conditions in October 2013 [5]: the subzone preparing for the lifting of the evacuation order (≤20 mSv/year), the restricted residential areas (20–50 mSv/year), and the subzone to which it was difficult to return (>50 mSv/year, after 5 years, the air dose rate was expected to be >20 mSv/year). The zoning of each site in the sampling period (including “outside the special decontamination areas”) was extracted from a zoning map of the special decontamination map [36] and treated as an ordinal variable with four levels. People in the subzone preparing for the lifting of the evacuation order were given relatively free access and could partly resume farming, although staying overnight was not allowed. People in the restricted residential areas were given relatively free access, although staying overnight and farming was not allowed. Access to and the performance of other activities in the subzone to which it was difficult to return were regulated. Decontamination by removal of soil has been gradually and patchily conducted, mainly in residential and agricultural areas in addition to the edges of forest; generally, priority has been given to areas with low radiation levels [37]. Thus, disturbances following usual anthropogenic activities were assumed to decrease with zoning level (i.e., the usual anthropogenic activities such as farming were the most frequent outside the evacuation zone).

To analyse the confounding effects of the landscape conditions before the earthquake, the following data were extracted for each site: the land cover area of the forests, paddy fields, and other agricultural lands and the land for buildings and rivers and lakes in 2009 [38]; the natural forest, secondary forest, and plantation forest based on a national vegetation survey conducted until 1998 [39]; the population in 2010 [4]; and the mean elevation and mean slope [40] within 1 × 1 km grid cells based on the “national standard mesh system” of Japan [41]. These GIS data were compiled using ArcGIS 10.2 (ESRI Japan Inc., Japan) and QGIS 2.2.0 [42].

Prior to the modelling study, the four-level zoning of the site was converted to three dummy variables, *E1* (within the subzone preparing for the lifting of the evacuation order), *E2* (within restricted residential areas), *E3* (within subzone to which it was difficult to return). To avoid multicollinearity, the other confounding environmental variables were analysed by Principal Component Analysis (PCA) and the four PCA scores, *PC1-4*, were obtained (S3 Table). The cumulative proportion of variance of the four scores exceeded 80%. Subsequently, the scores were standardised (centred and divided by the standard deviation). In addition, the position coordinates (in the UTM zone 54N) of the centre points of the *i*th school **s**_*i*_ = (*X*_*i*_, *Y*_*i*_) were obtained from national land numerical information download service [43].

### Statistical analyses

Bayesian regression models incorporating spatial autocorrelation [44] were constructed to compare the abundance patterns of each taxonomic group inside and outside the evacuation zone. We assumed that the effects of the evacuation zone were isotonic for the level of zoning (i.e., the regression coefficients of *E1*, *E2*, and *E3* were order-restricted) because the intensity of human intervention is expected to be negatively correlated with the zoning level. The number of individuals of a given taxonomic group *y*_*i*_ recorded in site *i* is assumed to follow a Poisson distribution, *y*_*i*_ ~ Poisson(*μ*_*i*_), where *μ*_*i*_ is the expected mean count in site *i*. The expected mean count is related to the linear predictors via the log-link function as:

$$log\left(\mu_{i}\right)=\;a_{0}+b_{0}\cdot (a_{1}\cdot E1_{i}+a_{2}\cdot E2_{i}+E3_{i})\;+b_{1}\cdot PC1_{i}+b_{2}\cdot PC2_{i}+b_{3}\cdot PC3_{i}+b_{4}\cdot PC4_{i}+\varepsilon_{i}$$

where *a*_*0*_ is the intercept, *b*_*0*_ is the regression coefficient for the dummy variable *E3*, and *a*_*1*_ and *a*_*2*_ are the positive down-weighting parameters for *E1* and *E2*, respectively, to incorporate the rank among the dummy variables (they must meet the constraint that *a*_*1*_ < *a*_*2*_ < 1). The parameters *b*_*1*_-_*4*_ are regression coefficients for *PC1*-*4*, respectively. *ε*_*i*_ is the spatial random effect by Bayesian kriging. Note that we focused on *b*_*0*_ as the coefficient for the effect of level of evacuation, which was expressed on the ordinal scale.

The specification of the model was determined to express the order restriction of the regression coefficients and spatial autocorrelation. To ensure constraint on *a*_*1*_, *a*_*2*_, we applied a broken-stick prior to these parameters using a uniform Dirichlet distribution, as follows:

$$a_{1}=p_{1}$$

and

$$a_{2}=p_{1}+p_{2},$$

where (*p*_1_, *p*_2_, *p*_3_) ~ Dirichlet(1, 1, 1). The vector of spatial random effect **ε** = (*ε*_1_, *ε*_2_, …) follows a multivariate normal distribution with a mean of 0 and a distance-dependent covariance matrix, as follows:

ε∼MVN(0,C/τ),

where *C*_*ij*_ = exp(-*φ*|**s**_*i*_—**s**_*j*_|). *τ* is the overall controlling parameter.

The model was fitted to the data with WinBUGS 1.4.3 and R2WinBUGS in R 3.0.3. For further details on the model and BUGS code, see S1 Panel. Model convergence was checked with R-hat values [45] and trace sites of the three chains for sampling [46].

## Supporting Information

S1 Data Analyzed insect data in Fukushima. (XLSX)

S1 Panel Bugs code for estimating an effect of exclusion level *E1-E3* (an ordinal-scale variable) on abundance of a taxonomic group *y*. Note that PCA scores are normalized by subtracting mean and dividing by SD for analysis.
(DOCX)

S1 Table Mean±S.D. abundance of each sampled taxonomic and caste group. (XLSX)

S2 Table Mean (upper row) ±S.D. (lower row) values of coefficients in the statistical Bayesian model. (XLSX)

S3 Table Eigen vectors for each PCA scores. (DOCX)
