# Supplementary material for: Pollinators and Other Flying Insects inside and outside the Fukushima Evacuation Zone
Source: PLoS One. 2015 Nov 11;10(11):e0140957. doi: 10.1371/journal.pone.0140957 (PMC4641662; doi:10.1371/journal.pone.0140957)
Supplement: S1 Panel — Note that PCA scores are normalized by subtracting mean and dividing by SD for analysis. (DOCX) [file pone.0140957.s002.docx]

Yoshioka et al. Pollinators and other flying insects inside and outside the Fukushima evacuation zone.

S1 Panel. Bugs code for estimating an effect of exclusion level *E1-E3* (an ordinal-scale variable) on abundance of a taxonomic group *y*. Note that PCA scores are normalized by subtracting mean and dividing by SD for analysis.

Prior distributions of parameters were set as non-informatively as possible. Normal distributions with mean of 0 and variance of 100 were used as prior distributions for a0, b0–4. Uniform distributions ranged from 0 to 100, and from 0.00001 to 0.01 were used as prior distributions for φ and σ (positive square root of inverse of τ), respectively.

Each MCMC algorithm was run with 3 chains with different initial values for 200000 iterations with the ﬁrst 100000 discarded as burn-in and the remainder thinned to one in every 100 iterations to save storage space (except for modelling of Diptera, *Crematogaster matsumurai*, *Camponotus japonicus*, which needs 700000 iterations with 200000 burn-in and 500 thinning for convergence).

The BUGS code is following:

model{

for(i in 1:N) {

yi[i]~dpois(mu[i])

log(mu[i])<-a0+b*z[i]+b1*PC1[i]+b2*PC2[i]+b3*PC3[i]+b4*PC4[i]+W[i]

z[i]<-a1*E1[i]+a2*E2[i]+E3[i]

muW[i]<-0

}

#Representing that a1 and a2 follows Dirichlet distribution using Conditional beta distribution.

a1~dbeta(1,2)

phi2~dbeta(1,1)

a2<- a1+(1-a1)*phi2

W[1:N]~spatial.exp(muW[],x[],y[],tau,phi,1)

a0~dnorm(0, 0.01)

b~dnorm(0, 0.01)

b1~dnorm(0, 0.01)

b2~dnorm(0, 0.01)

b3~dnorm(0, 0.01)

b4~dnorm(0, 0.01)

tau<-pow(sigma,-2)

sigma~dunif(0,100)

phi~dunif(0.00001,0.01)

}
