# Supplementary material for: Pollinators and Other Flying Insects inside and outside the Fukushima Evacuation Zone
Source: PLoS One. 2015 Nov 11;10(11):e0140957. doi: 10.1371/journal.pone.0140957 (PMC4641662; doi:10.1371/journal.pone.0140957)
Supplement: S3 Table — (DOCX) [file pone.0140957.s005.docx]

Yoshioka et al. Pollinators and other flying insects inside and outside the Fukushima evacuation zone.

Supplementary information

S3 Table. Eigen vectors for each PCA scores.

| Environmental variables | PC1 | PC2 | PC3 | PC4 |
| --- | --- | --- | --- | --- |
| Mean elevation | 0.3046 | -0.1076 | -0.4093 | 0.2205 |
| Mean slope | 0.4135 | 0.2223 | 0.0822 | -0.0128 |
| Population in 2010 | -0.3250 | 0.4435 | -0.2919 | -0.0814 |
| Paddy field area | -0.2248 | -0.4792 | 0.4227 | 0.0369 |
| Other agricultural land area | 0.0758 | -0.4764 | -0.5067 | -0.0623 |
| Forest area | 0.4295 | 0.1921 | 0.1103 | 0.0457 |
| Building area | -0.3655 | 0.3813 | -0.2621 | -0.0611 |
| Rivers and lakes area | -0.2553 | 0.1057 | 0.4056 | 0.1968 |
| Natural forest area | 0.1442 | 0.1958 | 0.1927 | -0.4540 |
| Secondary forest area | 0.3383 | 0.0445 | 0.1176 | -0.4781 |
| Plantation forest area | 0.2363 | 0.2279 | 0.0933 | 0.6782 |
